# Understanding diversity of human innate immunity receptors: analysis of surface features of leucine-rich repeat domains in NLRs and TLRs

**DOI:** 10.1186/1471-2172-10-48

**Published:** 2009-09-03

**Authors:** Andrei Y Istomin, Adam Godzik

**Affiliations:** 1 Burnham Institute for Medical Research, 10901 North Torrey Pines Rd., La Jolla, CA 92037, USA

## Abstract

**Background:**

The human innate immune system uses a system of extracellular Toll-like receptors (TLRs) and intracellular Nod-like receptors (NLRs) to match the appropriate level of immune response to the level of threat from the current environment. Almost all NLRs and TLRs have a domain consisting of multiple leucine-rich repeats (LRRs), which is believed to be involved in ligand binding. LRRs, found also in thousands of other proteins, form a well-defined "horseshoe"-shaped structural scaffold that can be used for a variety of functions, from binding specific ligands to performing a general structural role. The specific functional roles of LRR domains in NLRs and TLRs are thus defined by their detailed surface features. While experimental crystal structures of four human TLRs have been solved, no structure data are available for NLRs.

**Results:**

We report a quantitative, comparative analysis of the surface features of LRR domains in human NLRs and TLRs, using *predicted *three-dimensional structures for NLRs. Specifically, we calculated amino acid hydrophobicity, charge, and glycosylation distributions within LRR domain surfaces and assessed their similarity by clustering. Despite differences in structural and genomic organization, comparison of LRR surface features in NLRs and TLRs allowed us to hypothesize about their possible functional similarities. We find agreement between predicted surface similarities and similar functional roles in NLRs and TLRs with known agonists, and suggest possible binding partners for uncharacterized NLRs.

**Conclusion:**

Despite its low resolution, our approach permits comparison of molecular surface features in the absence of crystal structure data. Our results illustrate diversity of surface features of innate immunity receptors and provide hints for function of NLRs whose specific role in innate immunity is yet unknown.

## Background

The innate immune system provides a first level of defense against invading agents such as microbial pathogens and toxins and plays a role in maintaining a stable, healthy composition of the commensal microbiome. In mammals, the important role of continuously surveying the environment is played by members of two protein families: extracellular Toll-like receptors (TLRs) and intracellular Nod-like receptors (NLRs), also referred to as nucleotide-binding domain- and leucine-rich repeat-containing proteins. (In this manuscript we use NLR nomenclature as recommended by HUGO [1] as well as alternative names, such as NOD and NALP, which are more prevalent in current literature.) While individual receptors from both families have distinct specific roles, the overall function of the innate immune system requires their complex, synergistic interaction. (For recent reviews, see [2-4].)

Both TLR and NLR families belong to the oldest branch of the immune system, the innate immunity, and have complex evolutionary history that started in the simplest multicellular organisms and went through significant expansions and contractions in various lineages leading to present day vertebrates and mammals [5,6]. For instance, TLR and NLR families in some invertebrates, such as amphioxus, have over 80 members. In early branching vertebrates, such as sea lamprey and hagfish, a closely related family of variable lymphocyte receptors (VLRs) provide a version of adaptive immunity, which was lost in higher vertebrates [7].

In humans, the TLR family consists of ten proteins, which have been a subject of intense experimental studies during the past decade due to their critical role in immunity and involvement in diseases such as Crohn's disease [8], cystic fibrosis lung disease [9], inflammatory bowel disease [10], familial Mediterranean fever [11], septic joint disease [12], and several others. TLRs sense the presence of pathogen-associated molecular patterns (PAMPs) by specific binding sites in their extracellular "horseshoe"-shaped receptor domains consisting of multiple leucine-rich repeat (LRR) motifs. Binding-induced conformational change and/or dimerization provides a signal to the intracellular Toll/interleukin receptor (TIR) domain, which in turn binds specific adaptor molecules, such as Myd88, leading eventually to NF-κB transcription factor activation and transcription of proinflammatory cytokine genes [13]. From numerous experimental studies it is known that the LRR domains of different TLRs specifically interact with a rather diverse plethora of ligands, from bacterial flagellin and ss/dsRNA to peptidoglycans to imidazoquinioline compounds. By integrating these signals, the TLR receptors sense the microbial state of the immediate environment and define the appropriate level of innate immunity activity.

The NLR family receptors provide complementary, but less-studied and -understood, mechanisms of intracellular PAMP detection and innate immunity regulation. Twenty-two genes coding for NLR proteins are found in the human genome, typically consisting of a C-terminal LRR domain (homologous to the ligand-binding domain of TLRs), the nucleotide-binding oligomerization (NACHT) domain, and the N-terminal effector domain. Several different effector domains can be found in human NLRs, including the caspase recruitment (CARD) domain (in NOD1-4, IPAF, and CIITA); the pyrin/PAAD (PYD/PAAD) domain (in NALP1-14); the baculovirus "inhibitor of apoptosis" (BIR) domain (in NAIP); and an uncharacterized domain (in NLRX1), thereby partitioning NLRs into at least four subgroups. The variety of effector domains in other organisms, specifically invertebrates, is much larger [6]. Following binding of a ligand to the LRR domain, NALP1, NALP3, and IPAF are known to form multimer complexes termed "inflammasomes." Inflammasome formation leads to a further sequence of molecular events: activation of caspases and conversion of pro-interleukins into their active form. Recently, inflammasomes were shown [14] to be structurally similar to apoptosomes [15], highlighting the evolutionarily conserved mechanism of activated oligomerization, carried our by nucleotide binding AAA [16] proteins belonging to two distinct families: the NACHT family (PFAM PF05729, present in the NLR family) and NBARC family (PFAM PF00931, present in APAF family of apoptosis regulators).

After almost a decade since their discovery, information on ligand specificities of the NLR family proteins remains elusive. NOD1 and NOD2 are known to sense bacterial cell wall-derived peptidoglycans γ-D-glutamyl-meso-diaminopimelic acid (iE-DAP) [17] and muramyl dipeptide (MDP) [18,19], respectively. The NALP1 inflammasome was shown to be activated by MDP in a two-step manner [14]. The NALP3 inflammasome is activated by a wide range of ligands: bacterial RNA, uric crystals, antiviral imidazoquinioline compounds R837 and R848, and contact sensitizers (CSs) such as trinitro-chlorobenzene. NALP3 mutations are known to correlate with a number of autoinflammatory disorders, e.g., Muckle-Wells syndrome [20,21], and mutations in NOD2 gene are linked to increased susceptibility to the chronic inflammatory disorders such as Crohn's disease (CD), psoriatic arthritis, and Blau syndrome [22]. IPAF is known to sense bacterial flagellin [23,24], although whether flagellin is the direct and the only IPAF ligand is under debate [25]. Finally, CIITA that serves as a master regulator of major histocompatibility complex genes transcription is known to bind many DNA-binding and co-activator proteins. Its LRR domain was found to be involved in CIITA self-association [26] as well as in binding a novel zinc-finger protein ZXDC [27]. The specific roles of other NLRs in innate immunity (and/or in apoptosis) are at this point unknown. At the same time, there is no direct structure information on LRR domains of any of the NLRs. Given the importance of understanding NLRs' function and the lack of information on their structure and agonists, we believed a theoretical study might provide some useful insights and help design experimental efforts.

The main subjects of this work, the ligand-binding domains of NLRs and TLRs, both consist of multiple leucine-rich repeat (LRR) motifs. LRRs are extensively used by nature as building blocks for assembling scaffolds of protein interfaces, designed primarily for specific protein-protein interactions. About 500 LRR-containing proteins with diverse (and often unknown) functions have been identified in the human genome. Due to the importance of LRR proteins, they have been a subject of intensive experimental work, with the first crystal structure of a ribonuclease inhibitor [28,29] providing a major advance in the field. These advances continue today, with over 170 structures of LRR-containing proteins, including three TLR structures, solved through 2008. Much has been learned about structural principles of LRR proteins organization from theoretical and modeling perspectives [30-33]. Lastly, it is interesting to note that while LRR domains of TLR and NLR proteins are homologous, they belong to distinct sub-branches of the LRR family, and both have close homologs that are not innate immunity receptors. For instance, the families of LRR- and immunoglobulin-containing proteins, involved in cell-cell adhesion, are closer related to TLRs than NLRs are, and on the other hand, ribonuclease inhibitor, a protein not involved in innate immunity, is closely related to NLRs but is much more distant from TLRs. These observations suggest that NLR, TLR, and VLR families have a very complex evolutionary history with multiple duplication, domain swapping, and domain recruitment events.

While it is generally believed that LRR domains of most NLRs and TLRs are involved in direct binding of agonist molecules, for NLRs there is little experimental proof of this. Some evidence of direct agonist binding by NALP1 LRRs is given in [14]. When referring to "LRR ligands" in this manuscript, we recognize that binding to LRR domains is an assumption at this point, and that "ligands" can be either the "danger signals" themselves, or the possible intermediate molecules [34].

Since the overall shape of LRR domains from different proteins is relatively conserved, the functional similarities and differences between them must be defined by specific features of their surfaces. This would be true regardless of whether LRR domains interact with immunity "danger signals" directly or by means of intermediate molecules (e.g. similarly to TLR4 binding LPS-loaded MD2, or CIITA interacting with numerous transcription activator proteins), or the functionality is conferred by LRR interactions with other (e.g. NACHT or PYR) domains of NLRs. The key point is that if LRR surface properties within a given NLR/TLR pair are similar, they are likely to bind the broadly similar type of molecules.

In this manuscript, we investigate surface features of the twenty-two human NLRs, and by comparing them to those of TLRs, attempt to predict likely similarities between their functions. First, we have built homology models for LRR domains of twenty NLRs. Second, we have investigated distributions of amino acid charge and hydrophobicity across LRR domain surfaces and by means of clustering analysis have quantitatively assessed their similarities. Additionally, we have analyzed distributions of consensus N-linked glycosylation sites within TLRs, in order to support application of our approach to (presumably non-glycosylated) NLRs. While the simplicity of our analyses requires care when interpreting the results, we believe they may be helpful in designing future experiments to shed light on the role of NLR-coding genes in human innate immunity.

## Methods

### Phylogenetic analyses

**Figure 1:**
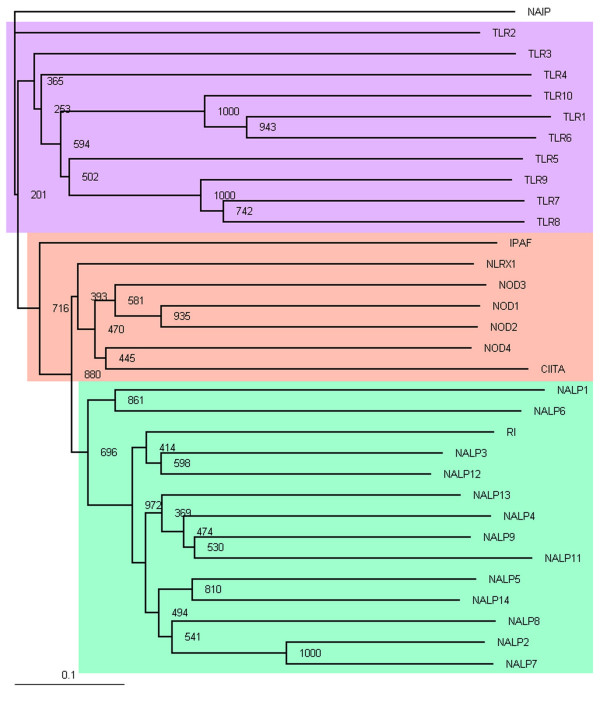
**Phylogeny of amino acid sequences of C-terminal LRR domains agrees with classification of innate immunity receptors according to their N-terminal effector domains**. The most divergent sequence, NAIP, was used to root the tree. Branch lengths are proportional to relative evolutionary distances. Integer numbers indicate bootstrap values (obtained by sampling over 1000 tree realizations) assessing statistical validity of the tree topology.

Multiple alignments and phylogenetic trees (see Figure 1) were built using ClustalX2 [35]. The most divergent sequence, NAIP, was used as an outgroup to root the tree. The tree reliability assessment was conducted by means of the bootstrap method using 1,000 tree samples. Several other multiple alignment and tree building strategies produced essentially identical results (data not shown). Protein sequences and gene organization information were obtained from the Ensembl database [36].

### Homology modeling

**Figure 2:**
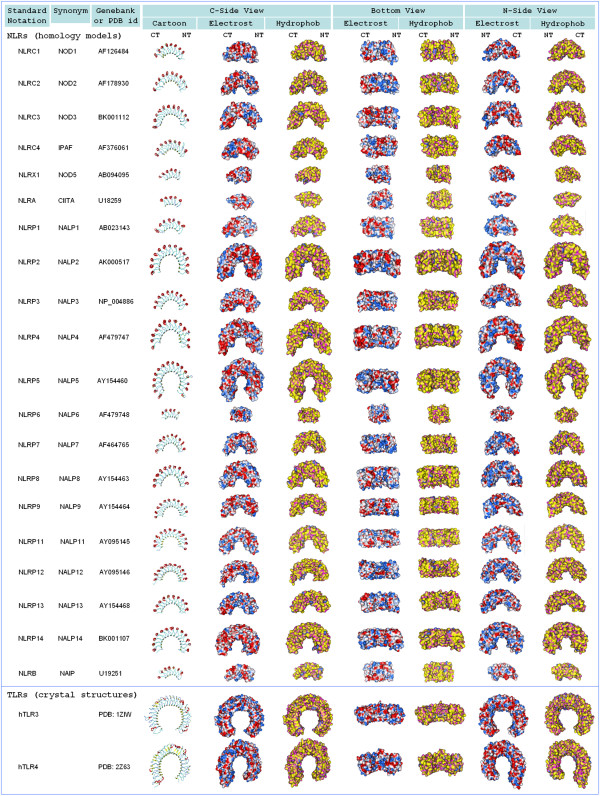
**Surface features of LRR domains from NLRs (homology models) and TLRs (X-ray crystal structures)**. Columns with three-dimensional structures show different views and representations of LRR domains. First column: cartoon representation colored according to secondary structure. Second, fourth, and sixth columns: molecular surfaces colored according to electrostatic potential. Third, fifth, and seventh columns: molecular surfaces colored according to hydrophobicity.

In order to visualize structures and surface features of LRR domains from human NLRs (see Figure 2), homology models were built based on pairwise alignments of NLR amino acid sequences with the sequence of porcine ribonuclease inhibitor (RI), whose X-ray structure (PDB id: 2BNH) was used as a template. The two proteins for which the models were not built are NOD4 and NALP10. NOD4 LRR domain consists of 43 LRRs, and all LRR-containing proteins with available structures are too small to be used as a template. The second protein, NALP10, lacks an LRR domain.

Amino-acid sequence alignments were manually refined using the Swiss-Pdb Viewer [37] to account for additional information obtained from the gene structure and from structural comparisons between LRR proteins (see below). Because of the high sequence similarity between the template and the prediction targets (50-60%), homology modeling was performed by means of the SWISS-MODEL server [38].

Electrostatics and hydrophobicity calculations were carried out for NLR homology models as well as for experimental crystal structures of two TLRs. They were conducted utilizing MOE software (Chemical Computing Group).

### LRR sequence-to-structure mapping

In order to conduct a quantitative comparative analysis of LRR domain surface features in the absence of experimental structure data, we mapped physicochemical features of residues on the surface using existing knowledge about LRR sequence-to structure-mapping [31-33].

The typical LRR sequence contains a conserved motif LxxLxLxxNxL, where L is leucine, isoleucine, valine, or phenylalanine, and N is asparagine, threonine, serine, or cysteine. In NLRs there is a striking correlation between their gene organization and amino acid sequence. Specifically, in NALPs, two consecutive LRR motifs XLaaNaLaaaaaaaaaaaaaaaaaLaaLXLaaNaLaaaaaaaaaaaaaaaaaaIaaL of length 28 + 29 = 57 residues are encoded by a single exon [3], while we have found that in NODs each LRR motif XLaaNaLaaaaaaaaaaaaaaaaaLaaL of length 28 residues is encoded by a single exon. This unusual genomic structure has interesting structural consequences, which would be a subject of a separate publication [39]. These two facts have allowed us to make reliable predictions for positions and boundaries of LRR motifs in human NLR sequences. In TLRs such an unusual structure-exon correlation is lacking, and the whole LRR domain is typically encoded by one large exon.

**Figure 3:**
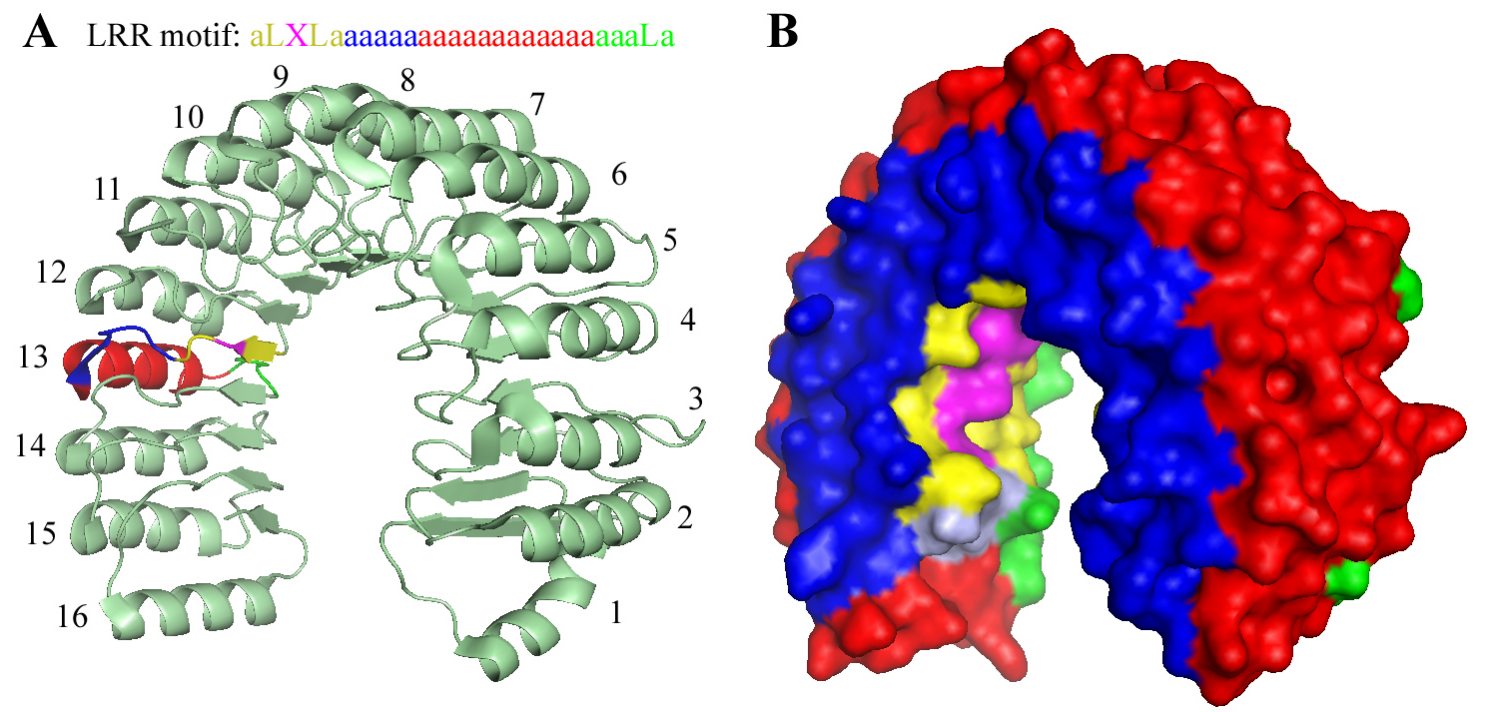
**An example of mapping the RI amino acid sequence into structure and of RI surface partitioning**. *A*, Mapping between an LRR sequence motif and the RI structure. RI sequence was searched for the conserved LRR pattern LaaLXL, and 16 LRRs were identified. The residue X (magenta) was then used as a reference to define residues belonging to the inner concave surface (yellow and magenta), N-terminal side (green), C-terminal side (blue), and outer convex surface (red). Mapping between a general LRR motif sequence and the structure of LRR #13 is shown by coloring. *B*, Full RI surface partitioned into four parts as described above.

Finally, from the available structures of other LRR-containing proteins it is known that the aaLXLaa pattern corresponds to a beta-strand in the inner concave surface, which is then followed by a C-terminal side loop, by an alpha-helix on the outside convex surface, and by the N-terminal side loop. This property is very well conserved in numerous LRR-containing proteins, and we have utilized it to improve mapping between LRR sequence and structure. The residue X in the aaLXLaa motif was used as reference residue to define positions of all other residues on the LRR surface (Figure 3A). The next 5 residues are located on the C-terminal side (usually loop conformation), the next 12 residues are located in the outer convex part (usually alpha-helical or loop conformation), and finally the next 5 residues represent the N-terminal side (usually loop conformation). In TLRs, the typical length of the outer part is 9 residues, but there is much variation.

After we performed the sequence-to-structure mapping as described above, we were able to partition the surface into four parts and analyze distributions of amino acid properties such as charge and hydrophobicity within these parts separately. Distribution of potential glycosylation sites was also examined for extracellular TLRs.

### Quantitative comparison of charge and hydropathy distributions

Amino acid charge and hydrophobicity distributions within LRR domains were calculated for each of the four surfaces shown in Figure 3B. The amino acid charge was calculated assuming the pH of 7.0, and the hydrophobicity was quantified by hydropathy index ranging from -4.5 to +4.5 [40] with negative (positive) values corresponding to hydrophilic (hydrophobic) properties.

Within a given protein, for each LRR motif *i*, we calculated average charge (*q*_*i*_) and hydropathy (*h*_*i*_) separately for each of the four surface regions. For an LRR domain with *n *motifs, this produced 4 *n *values for charge and the same for hydropathy. Next, in order to characterize the distribution of these values within each surface, we have calculated the first four moments, *m*_*k*_, (*k *= 0...3) of *q*_*i *_and *h*_*i *_distributions:

(1)

where *f*_*i *_is either *q*_*i *_or *h*_*i*_, and

(2)

With these definitions, the moment *m*_0 _is simply the average value, and the moment *m*_2 _is related to the square of the standard deviation. The moments *m*_1 _and *m*_3 _reflect additional details of the distributions, in particular the directional trends (N-terminus being more/less hydrophobic or charged that the C-terminus).

The distributions of charge and hydropathy within one protein are thus each characterized by a sequence of 4 surface × moments = 16 values. In order to quantify similarities of distributions within different proteins, we used Spearman's rank-order correlation coefficient, *r*_*S *_(it is similar to the usual Pearson's correlation coefficient, but reduces the effect of outliers). The similarity between distributions was then defined as (1 - *r*_*S*_). Clustering analysis was then performed by a complete linkage method utilizing Java Treeview software [41].

### Glycosylation distribution analysis

Consensus N-linked glycosylation sites were predicted by searching for patterns NxxS and NxxT in TLR sequences. In globular proteins, such predictions greatly overestimate the number of glycosylation sites, assuming that all sites matching the pattern above are accessible to oligosaccharyltransferases in order for glycosylation to occur, while in reality most of these sites are buried inside protein structure. In our case, however, such predictions can be much more accurate because we could predict which of the potential glycosylation sites were exposed to solvent.

## Results

### Phylogenetic analysis of NLR and TLR leucine-rich repeat domain sequences

As a first step, we have performed a phylogenetic analysis of LRR domains in NLRs and TLRs. Extreme sequence length diversity among LRRs from these families makes conducting such an analysis in a meaningful way nontrivial. This is further complicated by the fact that full length multiple alignment of complete sequences contains numerous large gap regions, which makes its truncation difficult. To circumvent the latter problem, we built a multiple alignment using a 10-fold increased gap extension penalty and truncated it from N- and C-terminal ends to make its length similar to that of the shortest sequence (see Additional File 1). The resulting alignment was then used to build a rooted phylogenetic tree (see Figure 1 and Methods). Among the NLR and TLR sequences, we also included a sequence of human ribonuclease inhibitor (RI) consisting of a highly-regular LRR domain closely homologous to that of LRRs in the NRL family.

The sequences group into three distinct clusters: (*i*) NALPs and RI; (*ii*) CARD-containing proteins and NLRX1; and (*iii*) TLRs. (Similar clustering was found when the multiple alignment was built using a default gap extension penalty and was used without truncation to infer a tree; data not shown.) Surprisingly, the sequence similarity-based phylogeny of the C-terminal LRR domain well agrees with the classification of all these proteins based on the N-terminal effector domain. Within NALPs, there are three distinct subgroups: (*a*) NALP2, NALP5, NALP7, NALP8, NALP14; (*b*) NALP4, NALP9, NALP11, NALP13; (*c*) NALP3, NALP12, and RI. It is interesting to note that IPAF and CIITA, as well as NLRX1 whose effector domain lacks definitive classification, group together with CARD-containing NODs. This suggests that NLRX1 effector domain might be CARD-like. This analysis supports known phylogenetic relationships between NLR and TLR families, but it does not permit us to draw any conclusions regarding similarities of surface features between TLRs with known ligands and NLRs.

### Homology models of LRR domains and sequence-to-structure mapping

In Figure 2 we present homology models for LRR domains of twenty NLRs (except NOD4 and NALP10) along with experimentally determined structures of two TLRs. The first observation is that there is much diversity in the LRR domain size - from 7 LRRs (in CIITA) to 19 LRRs (in NALP5). An unusually large number of LRRs (43 LRRs) is found in NOD4, whose model was not built because there is no large enough template structure available (see Discussion). Second, coloring of the models according to surface electrostatic potential and hydrophobicity reveals significant diversity of their surface features.

The models in Figure 2 are helpful in visualizing LRR domain structures and could be used for explicit protein-ligand docking; however they are not suitable for quantitative assessment of surface similarity. In order to quantitatively analyze the similarity of surface features of LRR domains, we resorted to their reduced-resolution representation. First, we adopted a simple approach to *implicitly *infer LRR domain three-dimensional structures from their amino acid sequences (see Figure 3A and Methods). Second, we have partitioned the total surface of the LRR domain of each protein into four regions (see Figure 3B and Methods): concave inner surface (mainly beta-strands), N-terminal side, C-terminal side, and convex outer surface (mainly alpha-helices in NLRs and RI, and loops or beta strands in TLRs). Before conducting a quantitative comparison of NLR and TLR surface features we had to consider possible perturbations to TLR surface properties due to glycosylation.

### Role of glycosylation of LRR domain surfaces

It is well known that LRR domains in all ten human TLRs are N-glycosylated due to their extracellular localization, and in some of them glycosylation is essential for proper function [42,43]. This is not surprising as glycosylation can dramatically alter surface properties and thereby affect ligand binding. Since NLRs are all known to be cytosolic proteins their N-linked glycosylation is unlikely, which complicates a direct comparison of their surface features to those of TLRs. To understand the potential role of TLR glycosylation in their ligand binding, we have performed a search for consensus N-linked glycosylation sites within TLR sequences, and analyzed their distribution over the domain surfaces. The results are summarized in Table 1.

**Table 1:** Predicted numbers of N-linked consensus glycosylation sites (NxT, NxS) in TLR ectodomains for each of the four domain surfaces.

**Protein**	**Number of consensus glycosylation sites**			
	**inner**	**N-side**	**C-side**	**outer**
TLR1	4	1	0	0
TLR2	1	2	0	1
TLR3	3	7	0	6
TLR4	1	3	0	0
TLR5	2	2	1	3
TLR6	3	0	1	2
TLR7	3	1	1	6
TLR8	3	4	0	7
TLR9	4	4	1	6
TLR10	2	1	1	1

The TLRs that contain many consensus glycosylation sites are TLR3, TLR7, TLR8, and TLR9. In TLR3, all surfaces except the C-terminal side that is known to bind dsRNA contain several glycosylation sites. In TLR7, inner and outer surfaces are likely to be glycosylated while side ones are not, which is consistent with binding of ssRNA via one of its side surfaces. Glycosylation of TLR8 and TLR9 resembles that of TLR3 in that only the C-terminal side surface is glycan-free. In the rest of the TLRs, each of the four surfaces contains no more then three potential glycosylation sites. As an additional step, we have also analyzed NLR sequences for potential glycosylation (data not shown), and found that they contain 0-2 consensus glycosylation sites per surface, with the only exception being an N-terminal surface of NOD3, which interestingly contains 10 such sites.

These findings indicate that while glycosylation of some TLRs certainly modulates their surface properties, in most cases the ligand binding sites are located in non-glycosylated regions and, in addition, the overall surface area affected by glycosylation is relatively small, so that global surface comparisons such as those performed here are not affected.

### Hydrophobicity and charge distributions: clustering analysis within full LRR surfaces

**Figure 4:**
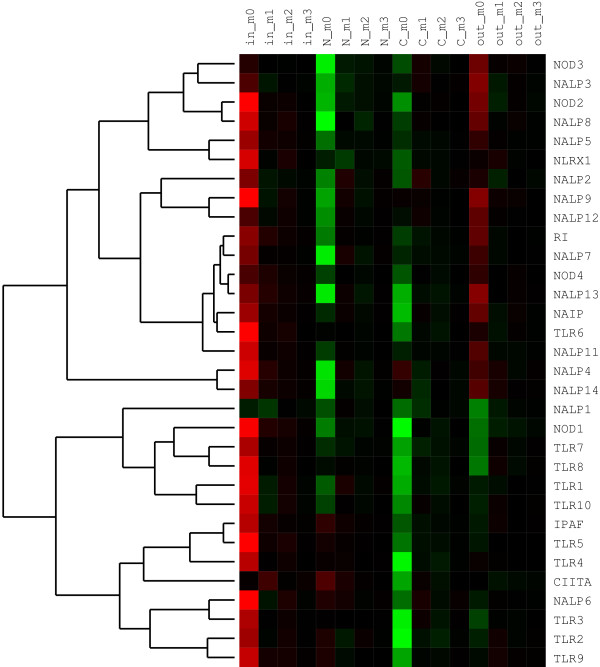
**Hierarchical clustering analysis of amino acid hydropathy distributions within LRR domains of NLRs and TLRs**. Color matrix shows values for first four moments (m_0_,..., m_3_; cf. Equation 1) of the hydropathy distribution over four LRR domain surfaces: inner (concave), N-terminal side, C-terminal side, and outer (convex). Red coloring corresponds to positive values of the moments, black to zero, and green to negative values. The measure of similarity between sequences of moment values of LRR surfaces is the Spearman's rank-order correlation coefficient, *r*_*S*_. The tree on the left is the result of hierarchical clustering of pairwise distances by the complete linkage method. Length of edges is proportional to distances, *d*_*S*_, between sequences of moment values and is defined as *d*_*S *_= 1 - *r*_*S*_. Grouping of LRR domains into clusters indicates overall similarity of their hydrophobicity distributions.

The two physico-chemical amino acid properties that critically affect ligand binding and whose distributions we have thus analyzed are charge and hydrophobicity. Figure 4 shows an example of our clustering analyses of hydropathy distribution moments within each of the four domain surfaces. The similarity measure in this analysis is Spearman's correlation (see Methods). Based on clustering results, LRR domain pairs that have particularly similar hydropathy distributions and LRR numbers are: TLR5 and IPAF (both respond to bacterial flagellin, although for IPAF this is under debate [25]); NOD2 (ligand: MDP) and NALP8 (ligand unknown); RI (ligand RNase) and NALP7 (ligand unknown); TLR6 (ligand: diacyl lipopeptide) and NAIP (ligand unknown); NALP4 (ligand unknown) and NALP14 (ligand unknown); TLR1 (ligand: triacyl lipopeptide) and TLR10 (ligand unknown); TLR3 (ligand: dsRNA) and NALP6 (ligand unknown).

**Figure 5:**
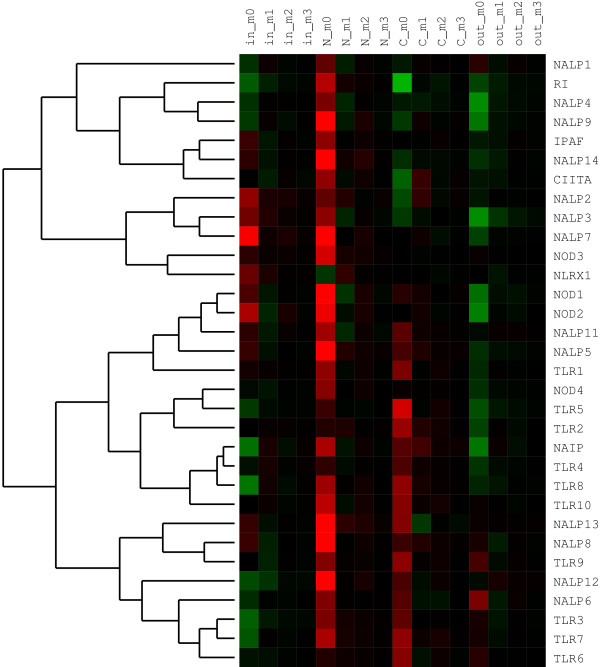
**Hierarchical clustering analysis of amino acid charge distributions within LRR domains of NLRs and TLRs**. All notations are the same as in Figure 4.

Figure 5 presents results of a similar clustering analysis of charge distributions. The domain groups with similar hydrophobicity distributions are: RI (ligand: RNase) and NALP9 (ligand unknown); NALP3 (ligands: RNA, small molecules) and NALP2 (ligand unknown); TLR8 (ligands: ssRNA, imidazoquiniolines) and NAIP; TLR3 (ligand: dsRNA), TLR7 (ligands: ssRNA, small molecules), and NALP6 (only 8 LRRs; ligand unknown).

### Hydrophobicity and charge distributions within individual LRR domain surfaces

In order to gain further insight into similarities of LRR surface features between NLRs and TLRs, we conducted a careful manual inspection of similarity patterns across individual biologically relevant surfaces, also taking into account results of our TLR glycosylation analysis (cf. Table 1). We assessed similarities between LRR domains of all 32 proteins considered, as detailed below. (*i*) In NALP2, which is known to sense MDP, the concave and side surfaces have both hydropathy and charge distributions that are the most similar to NAPL3, which is known to bind a diverse set of ligands, including MDP. (*ii*) NALP4 and NALP14 have similar hydropathy distributions, but dissimilar charge distributions. (*iii*) NALP5 and NALP8 have hydropathy and charge distributions similar to NOD1 (except over the outer convex surface) that binds iE-DAP. (*iv*) NALP6 has the shortest LRR domain, containing only 6 LRRs. Its surface properties are similar to those of TLR3, which contains 25 LRRs. Because the hydropathy and charge distribution moments are normalized by the number of LRRs in a given domain, similarity of distributions still allows to hypothesize that NALP6 and TLR3 may bind the same type of ligands, RNA motifs. (*v*) NALP7 charge and hydropathy are similar to NOD2 and NALP3 both binding MDP, suggesting small molecules as probable ligands. (*vi*) The high similarity of NALP8 hydropathy distribution and size to those of NOD2 and also NOD1 (whose convex surface hydropathy is different) suggests that they may share the same type of ligands--peptidoglycans. This is also supported by similarities in their charge distributions, with the difference being a more positively charged convex surface of NALP8. (*vii*) The hydropathy and charge of NALP9 are similar to RI, which binds RNase, thus suggesting that the NALP9 ligand may be protein-like. (*viii*) NALP11 shows hydropathy similarity with TLR7 that binds ssRNA, most likely by one of its side surfaces. NALP11 charge distribution is similar to that of TLR7 and also TLR3 (which binds dsRNA at its side surface) over its side surfaces, suggesting that ss/dsRNA is likely to be among possible NALP11 ligands. (*ix*) NALP12 surface properties do not exhibit any obvious similarity to others. (*x*) The hydropathy distribution of NALP13 is similar to that of NALP7 and NOD4, but neither of them has known ligands. (*xi*) NALP14 charge distribution on its concave surface is almost identical to that of IPAF, and distributions over other surfaces are similar to IPAF. The hydropathy distributions of these two NLRs are similar within only its concave side. Assuming that IPAF binds flagellin similar to TLR5, i.e., on its concave surface [44], flagellin can be considered a likely ligand of NALP14. (*xii*) NOD3 hydropathy has some similarity to NALP3, but charge distribution is unlike that in any other domain. (*xiii*) NOD4 concave and side surfaces have hydrophobicity similar to NALP12, but the charge distribution is different. Also, NOD4 appears to have the largest ligand-binding domain of all NLRs and TLRs: it contains as many as 43 LRRs. (*xiv*) NLRX1 surface features do not exhibit similarity with any other domain. (*xv*) NAIP hydropathy distribution is similar to RI, while NAIP charge distribution is similar to TLR8.

It is interesting to note that the functional similarities inferred based on comparison of molecular surfaces correlate well with experimental data where available, while hypotheses based on evolutionary relationships shown in Figure 1 generally lack such correlation. For example, while the surface comparison and the known experimental data suggest that NALP2 and NALP3 are activated by the same type of molecules, MDP, the phylogenetic reconstruction places NALP2 and NALP3 into two distinct subgroups in Figure 1, although both subgroups belong to the NALP group. Similarly, whereas IPAF and TLR5 are known from experiment to sense a common molecule (bacterial flagellin) with which our surface analysis is in agreement, in the phylogenetic analysis they are placed into two distinct groups. The closest agreement between surface-based and phylogeny-based functional inferences appears to be between NALP5 and NALP8.

The above surface similarity considerations, which we believe are the most interesting results of this work, are summarized in Table 2. For each NLR with unknown ligands, we indicate to which other TLRs/NLRs its charge and hydropathy distributions are the most similar, and based on this infer the molecules that are likely to be its ligands. We would like to stress that because of low resolution of our approach, the suggested similarity-based predictions for putative NLR ligands should be considered with care.

**Table 2:** Summary of putative NLR ligands predicted to bind to their LRR domains, based on similarity of surface features between NLRs and TLRs with known agonists.

**HUGO name**	**Nomenclature**	**Known agonists**	**Hydropathy similarity**	**Charge similarity**	**Putative ligands**
**NOD1**	**NOD1**	**iE-DAP**	NALP5, NALP8	**NOD2**, NALP5, NALP8	n/a
**NOD2**	**NOD2**	**MDP**, also modulates NALP1 activation	NALP7, NALP8	**NOD1**, NALP7, NALP8	n/a
NLRC3	NOD3		NALP3		no prediction
**NLRC4**	**IPAF**	**Bacterial flagellin**	**TLR5**, NALP14, CIITA	NALP14	n/a
NLRC5	NOD4		NALP12, NALP13		no prediction
NLRP1	NALP1	MDP			n/a
**NLRP2**	**NALP2**	**MDP**	**NALP3**	**NALP3**	n/a
**NLRP3**	**NALP3**	Bacterial RNA, **MDP**, R837, R848, CSs, etc.	**NALP2**, NOD3	**NALP2**, NALP7	n/a
NLRP4	NALP4		NALP14		no prediction
NLRP5	NALP5		NOD1, NALP8	NOD1, NALP8	small molecules
NLRP6	NALP6		TLR3	TLR3	RNA/DNA
NLRP7	NALP7		NOD2, NALP13	NOD2, NALP3	small molecules
NLRP8	NALP8		NOD1, NOD2, NALP5	NOD1, NOD2, NALP5	small molecules
NLRP9	NALP9		RI	RI	protein-like
NLRP10	NALP10				no prediction
NLRP11	NALP11		TLR7	TLR3, TLR7	RNA
NLRP12	NALP12	Antagonizes IRAK-1	NOD4		no prediction
NLRP13	NALP13		NOD4, NALP7		no prediction
NLRP14	NALP14		NALP4, IPAF	IPAF	flagellin
NLRB1	NAIP		RI	TLR8	no prediction
NLRX1	NOD5				no prediction
**NLRA**	**CIITA**	**ZXDC binds to LRR region**	**IPAF, TLR4, TLR5**		protein-like
	RI	Ribonuclease	NALP9, NAIP	NALP9	n/a
	TLR1	triacyl lipopeptide (in complex with TLR2)			n/a
	TLR2	triacyl-, diacyl-lipopeptides (in complex with TLR1, TLR6)			n/a
	TLR3	dsRNA	NALP6	NALP6, NALP11	n/a
	TLR4	LPS-loaded MD2	CIITA		n/a
	**TLR5**	**Bacterial flagellin**	**IPAF**, CIITA		n/a
	TLR6	diacyl-lipopeptide (in complex with TLR2)			n/a
	**TLR7**	**ssRNA (HIV, SSV, influenza), imidazoquiniolines (e.g., R837), loxoribrine**	**TLR8**, NALP11	NALP11	n/a
	**TLR8**	**ssRNA, imidazoquiniolines (e.g., R848)**	**TLR7**	NAIP	n/a
	TLR9	Bacterial and viral CpG DNA motifs			n/a
	TLR10				no prediction

Cases with correctly reproduced similarities are highlighted in bold. In the "putative ligands" column, cases with known ligands are denoted by "n/a", and cases where no prediction was made are marked accordingly.

## Discussion

In this paper we use similarities between surface features of two families of innate immunity receptors to reason about their possible functional similarities. The validity of this approach is supported by results presented here - for instance, IPAF and TLR5, which despite distant phylogenetic relationship both respond to bacterial flagellin (regarding IPAF, see [25]), show very similar distribution of hydrophobic surface residues (cf. Figure 4). Other examples include similarities in binding specificities and hydrophobic distribution moments between TLR7 and TLR8 (both sense RNA), between CIITA and TLR5 (both respond to protein-like targets), similar charge distribution between NOD1 and NOD2 (both respond to similarly charged iE-DAP and MDP, respectively), and between NALP2 and NALP3 (both sense MDP) (cf. Figure 5).

These correlations suggest that surface similarities between NLRs and TLRs found here may reflect possible but as yet unknown functional similarities. For most NLR genes the binding specificities are unknown, and only indirect functional information, such as from RNA and protein expression profiles, is available [45]. In the following, we have attempted to put the possible predicted functional similarities within NLR families (cf. Table 2) into the context of what is known from such experimental studies. We realize that the questions asked in most of those studies have quite distant relation to the NLR molecular surface features we focus on here, therefore such a comparison rarely allows for definitive conclusions.

Specific cases of similarities found here include predicted functional similarity between NALP4 and NALP14, but this doesn't allow for any inferences as the ligands for both are unknown; it is only known that NALP14 plays a role in spermatogenesis and its expression is restricted to testis [46]. The similarity between NALP5 and NALP8 indirectly correlates with the fact that their transcripts are both detected in reproductive organs (ovaries and ovaries/testis, respectively [45]), but again neither of their binding specificities is known. Their surface similarity to NOD1 indicates small molecules as potential binding partners, but in contrast to MDP it is unlikely that they are bacteria-derived. It should be noted that NALP5 was also implicated as an autoantigen in autoimmune polyendocrine syndrome type 1 [47], and also that transient expression of recombinant NALP1 or NALP5 in neurons was found to induce caspase-3 activation and apoptosis [48]. NALP6 (as well as NALP12) were suggested to play a role in immunity by activating NF-κB signaling and caspase-1-dependent cytokine processing [49,50]. Here we find similarity between the very short NALP6 LRR domain and the TLR3, which suggests NALP6 may respond to RNA motifs.

An interesting observation is that NALP7, which is implicated in recurrent hydatidiform moles and reproductive wastage in humans and is known to inhibit IL-1beta upon stimulation with such small molecules as LPS *in vitro *[51], appears in our analysis to have surface features similar to NOD2 that binds MDP. On the contrary, NALP9, whose expression profiles indicate its localization to testis and ovary similarly to NALP5 and NALP8, in our analysis displayed most significant similarity to RI, which implies other proteins as possible binding partners. NALP10 lacking the LRR domain was excluded from the analyses. NALP11 showed charge distribution similarity with TLR3 and TLR7 implying potential to bind RNA, but we are unaware of any reports on related experimental studies. NALP12 has been shown to suppress the non-canonical NF-κB pathway by inducing degradation of NIK (NF-κB inducing kinase) [52], but its ability to form an "inflammasome" still awaits experimental proof. In our analysis NALP12 surface properties did not display similarity to any other proteins. Similarity of NALP13 to NALP7 and to NOD4 did not allow us to draw any functional similarity inferences, and we are unaware of experimental studies of this receptor. The last member of the NALP family, NALP14, displays a controversial similarity to IPAF because it implies bacterial flagellin as potential ligand, while NALP14 expression is restricted to testis [46]; this however may point to NALP14 potential role of responding to other proteins.

Surface features of NOD3, NOD4, and NLRX1 did not provide us with an insight to make functional inferences. Two observations however need to be mentioned here. First, NOD3 contains unusually large number (as many as 10) of potential N-linked glycosylation sites on its N-terminal side, unlike in any other NLR and TLR. This however likely has little relevance to possibility of actual NOD3 glycosylation, as currently there are no reports of it being secreted. Second, as mentioned above, NOD4 appears to have extremely large number of LRRs (as many as 43) making its LRR domain the largest among all NLRs and TLRs. This implies that the NOD4 LRR domain structure may possess some of the following properties untypical for NLRs: (*i*) in-plane circular shape with very large radius (i.e., low curvature); (*ii*) nonplanar, torroid-like shape with regular radius (normal curvature); (*iii*) structure consists of two or more connected circular parts each having regular radius/curvature. The actual three-dimensional shape of this domain thus represents a puzzle. Lastly, NAIP surface properties resemble those of RI and TLR8 binding ribonuclease and RNA, respectively. Murine Naip5 (one of seven paralogue genes) was shown to be required for intracellular response to bacterial flagellin in mice [53], which indirectly signifies NAIP's potential for binding protein fragments.

Results in Figures 4 and 5 also point to a significant diversity of LRR domain surface features within both TLR and especially NLR families. This diversity is apparent even given the low resolution of our analyses. We believe this is mostly a consequence of the need of the receptors to recognize a group of very diverse ligands (from small molecules to protein/RNA/DNA fragments) and their binding modes.

Finally, in NLRs the interactions between CARD/PYR/BIR, NACHT, and LRR domains are likely to play important roles in their oligomerization and activation. The relative three-dimensional orientation of the LRR, NACHT, and effector domains is likely to vary within the NLR family, which is signified e.g. by generally large and highly variable size of the linker domain (connecting the NACHT and LRR domains). We believe that this could be another possible explanation of the diversity of LRR domain surface properties within NLRs, which in turn points to the possibility of diverse ligand-induced inflammasome activation mechanisms.

## Conclusion

We have built homology models and performed quantitative analyses of charge, hydrophobicity, and glycosylation distributions of LRR domains in almost all human NLRs and TLRs known today. These analyses, while conducted without experimental knowledge of NLR structures, provide a low-resolution comparison of LRR surface features between the two protein families. The validity of our approach is supported by several cases when receptors that bind similar ligands were found to have similar charge and hydrophobicity distributions. Comparison of NLRs with unknown functions to NLRs/TLRs that have known ligands allowed us to infer possible functional similarities, which can not be derived from conventional phylogenetic analyses. We hope that our predictions will be helpful in design of future experiments to decipher roles of previously uncharacterized NLRs in human innate immunity. We propose an experimental study to test whether the NLR macromolecular ligands we predict indeed modulate the activity of caspase-1 in the presence and absence of the corresponding NLRs *in vitro*. Because of the low resolution of our approach, our predictions should be more reliable in cases where TLR/NLR known ligands are macromolecular fragments, such as flagellin, ds/ssRNA, or DNA motifs. Improving the reliability of predictions and providing quantitative estimates for relative ligand binding affinities requires combined application of explicit structure-based approaches such as homology modeling and molecular docking. Work in this direction is in progress and will be reported elsewhere.

## Authors' contributions

AYI performed the calculations, analyzed the results, and drafted the manuscript. AG helped to formulate and coordinated the study, and edited the manuscript. Both authors read and approved the final manuscript.

## Supplementary Material

Additional file 1 **Supplementary materials**. A multiple sequence alignment used for phylogenetic analysis in Figure 1. Click here for file
