# Supplementary material for: Understanding diversity of human innate immunity receptors: analysis of surface features of leucine-rich repeat domains in NLRs and TLRs
Source: BMC Immunol. 2009 Sep 3;10:48. doi: 10.1186/1471-2172-10-48 (PMC2747839; doi:10.1186/1471-2172-10-48)

SUPPLEMENTARY MATERIALS

Understanding Diversity of Human Innate Immunity Receptors: Analysis of Surface Features of Leucine-Rich Repeat Domains in NLRs and TLRs

Andrei Y. Istomin* and Adam Godzik*

*Burnham Institute for Medical Research, 10901 North Torrey Pines Rd, La Jolla, CA 92037

Contents

1. Multiple sequence alignment used for phylogenetic analysis in Figure 1 . . . . p. 3

```
NOD1     ELQPCFS----RLTVLRLSVNQITDGGVKVLSEELTKYKIVTYLGLYNNQITDVGARYVTKILDECKG-LTHLKLGKNK---ITSEGGKYLALAVKNSKSISEVGMWGNQVGDEGAKAFAEALRNHP-SLTTLSLASNGISTEGGKS---   138
NOD2     QLLPCLG----VCKALYLRDNNISDRGICKLIECALHCEQLQKLALFNNKLTDGCAHSMAKLLACRQN-FLALRLGNNY---ITAAGAQVLAEGLRGNTSLQFLGFWGNRVGDEGAQALAEALGDHQ-SLRWLSLVGNNIGSVGAQA---   138
NOD3     RMADLKQN-R-SLKELMFSSNSIGDGGAKALAEALKVNQGLESLDLQSNSISDAGVAALMGALCTNQT-LLSLSLRENS---ISPEGAQAIAHALCANSTLKNLDLTANLLHDQGARAIAVAVRENR-TLTSLHLQWNFIQAGAAQA---   140
NOD4     HLASGLGH-CHHLEELDLSNNQFDEEGTKALMRALEGKWMLKRLDLSHLLLNSSTLALLTHRLSQMTC-LQSLRLNRNS---IGDVGCCHLSEALRAATSLEELDLSHNQIGDAGVQHLATILPGLP-ELRKIDLSGNSISSAGGVQ---   141
NLRX1    ------------LDEVNLASCQLDPAGLRTLLPVFLR---ARKLGLQLNSLGPEACKDLRDLLLHDQCQITTLRLSNNP---LTAAGVAVLMEGLAGNTSVTHLSLLHTGLGDEGLELLAAQLDRNR-QLQELNVAYNGAGDTAALA---   128
NALP3    VLCETLQHPGCNIRRLWLGRCGLSHECCFDISLVLSSNQKLVELDLSDNALGDFGIRLLCVGLKHLLCNLKKLWLVSCC---LTSACCQDLASVLSTSHSLTRLYVGENALGDSGVAILCEKAKNPQCNLQKLGLVNSGLTSVCCSA---   144
NALP12   LLCEGLRHPQCRLQMIQLRKCQLESGACQEMASVLGTNPHLVELDLTGNALEDLGLRLLCQGLRHPVCRLRTLWLKICR---LTAAACDELASTLSVNQSLRELDLSLNELGDLGVLLLCEGLRHPTCKLQTLRLGICRLGSAACEG---   144
RI       LLCEGLLDPQCRLEKLQLEYCSLSAASCEPLASVLRAKPDFKELTVSNNDINEAGVRVLCQGLKDSPCQLEALKLESCG---VTSDNCRDLCGIVASKASLRELALGSNKLGDVGMAELCPGLLHPSSRLRTLWIWECGITAKGCGD---   144
NALP9    HLCETLKHPMCKIEELILGKCDISSEVCEDIASVLACNSKLKHLSLVENPLRDEGMTLLCEALKHSHCALERLMLMYCC---LTSVSCDSISEVLLCSKSLSLLDLGSNALEDNGVASLCAALKHPGCSIRELWLMGCFLTSDSCKD---   144
NALP11   LLHDILHEPTCQISHLSLMKCDLRASECEEIASLLISGGSLRKLTLSSNPLRSDGMNILCDALLHPNCTLISLVLVFCC---LTENCCSALGRVLLFSPTLRQLDLCVNRLKNYGVLHVTFPLLFPTCQLEELHLSGCFFSSDICQY---   144
NALP4    SLCDALNYPAGNVKELALVNCHLSPIDCEVLAGLLTNNKKLTYLNVSCNQL-DTGVPLLCEALCSPDTVLVYLMLAFCH---LSEQCCEYISEMLLRNKSVRYLDLSANVLKDEGLKTLCEALKHPDCCLDSLCLVKCFITAAGCED---   143
NALP13   LILKALRHSACNLKYLCLEKCNLSAASCQDLALFLTSIQHVTRLCLGFNRLQDDGIKLLCAALTHPKCALERLELWFCQ---LAAPACKHLSDALLQNRSLTHLNLSKNSLRDEGVKFLCEALGRPDGNLQSLNLSGCSFTREGCGE---   144
NALP5    MACEALKHPKCLLESLRLDCCGLTHACYLKISQILTTSPSLKSLSLAGNKVTDQGVMPLSDALRVSQCALQKLILEDCG---ITATGCQSLASALVSNRSLTHLCLSNNSLGNEGVNLLCRSMRLPHCSLQRLMLNQCHLDTAGCGF---   144
NALP14   SLCEALKHPECKLQTLRLESCNLTVFCCLNISNALIRSQSLIFLNLSTNNLLDDGVQLLCEALRHPKCYLERLSLESCG---LTEAGCEYLSLALISNKRLTHLCLADNVLGDGGVKLMSDALQHAQCTLKSLVLRRCHFTSLSSEY---   144
NALP2    ALCEVLRHPECNLRYLGLVSCSATTQQWADLSLALEVNQSLTCVNLSDNELLDEGAKLLYTTLRHPKCFLQRLSLENCH---LTEANCKDLAAVLVVSRELTHLCLAKNPIGNTGVKFLCEGLRYPECKLQTLVLWNCDITSDGCCD---   144
NALP7    MLCDLLRNHKCNLQYLRLGGHCATPEQWAEFFYVLKANQSLKHLRLSANVLLDEGAMLLYKTMTRPKHFLQMLSLENCR---LTEASCKDLAAVLVVSKKLTHLCLAKNPIGDTGVKFLCEGLSYPDCKLQTLVLQQCSITKLGCRY---   144
NALP8    LLCRVLRSPRCRLQCLRLEDCLATPRIWTDLGNNLQGNGHLKTLILRKNSLENCGAYYLSVAQ------LERLSIENCN---LTQLTCESLASCLRQSKMLTHLSLAENALKDEGAKHIWNALPHLRCPLQRLVLRKCDLTFNCCQD---   138
TLR1     TTWNSFIRILQLVWHTTVWYFSISNVKL--QGQLDFRDFDYSGTSLKALSIHQVVSDVFGFPQSYIYEIFSNMNIKNFT---VSGTRMV------HMLCPSKISPFLHLDFSNNLLTDTVFENCGHLTELETLILQMNQLKELSKIA---   136
TLR6     TTWKCLVRVFQFLWPKPVEYLNIYNLTI--IESIREEDFTYSKTTLKALTIEHITNQVFLFSQTALYTVFSEMNIMMLT---ISDTPFI------HMLCPHAPSTFKFLNFTQNVFTDSIFEKCSTLVKLETLILQKNGLKDLFKVG---   136
TLR10    LLWDDLFLILQFVWHTSVEHFQIRNVTFGGKAYLDHNSFDYSNTVMRTIKLEHVHFRVFYIQQDKIYLLLTKMDIENLT---ISNAQMP------HMLFPNYPTKFQYLNFANNILTDELFKRTIQLPHLKTLILNGNKLETLSLVS---   138
TLR7     QTLDLSKNSIFFVKSSDFQHLSFLKCLNLSGNLISQTLNGSEFQPLAELRYLDFSNNRLDLLHSTAFEELHKLEVLDIS---SNSHYFQSEGITHMLNFTKNLKVLQKLMMNDNDISSSTSR-TMESESLRTLEFRGNHLDVLWREGD--   144
TLR8     KALDLSLNSIFFIGPNQFENLPDIACLNLSANSNAQVLSGTEFSAIPHVKYLDLTNNRLDFDNASALTELSDLEVLDLS---YNSHYFRIAGVTHHLEFIQNFTNLKVLNLSHNNIYTLTDKYNLESKSLVELVFSGNRLDILWNDDD--   145
TLR9     FTLDLSRNNLVTVQPEMFAQLSHLQCLRLSHNCISQAVNGSQFLPLTGLQVLDLSHNKLDLYHEHSFTELPRLEALDLS---YNSQPFGMQGVGHNFSFVAHLRTLRHLSLAHNNIHSQVSQ-QLCSTSLRALDFSGNALGHMWAEG---   143
NALP1    HLCQRLRQPSCKLQRLQLVSCGLTSDCCQDLASVLSASPSLKELDLQQNNLDDVGVRLLCEGLRHPACKLIRLGLDQTT---LSDEMRQELRALEQEKPQLLIFSRRKPSVMTPTEGLDTGEMSNSTSSLKRQRLGSERAASHVAQAN--   145
CIITA    ------------------VQTQRTRSSSEDTAGELPAVRDLKKLEFALGPVSGPQAFPKLVRILTAFSSLQHLDLDALSENKIGDEGVSQLSATFPQLKSLETLNLSQNNITDLGAYKLAEALPSLAASLLRLSLYNNCICDVGAES---   129
TLR3     WLKCLEHLNMEDNDIPGIKSNMFTGLINLKYLSLSNSFTSLRTLTNETFVSLAHSPLHILNLTKNKISKIESDAFSWLGHLEVLDLGLNEIGQELTGQEWRGLENIFEIYLSYNKYLQLTRNSFALVPSLQRLMLRRVALKNVDSSP---   147
NALP6    PLFQAMTDPLCHLSSLTLSHCKLPDAVCRDLSEALRAAPALTELGLLHNRLSEAGLRMLSEGLAWPQCRVQTVRVQLPD----PQRGLQYLVGMLRQSPALTTLDLSGCQLPAPMVTYLCAVLQHQGCGLQTLSLASVELSEQSLQE---   143
TLR4     DKSALEGLCNLTIEEFRLAYLDYYLDDIIDLFNCLTNVSSFSLVSVTIERVKDFSYNFGWQHLELVNCKFGQFPTLKLK------SLKRLTFTSNKGGNAFSEVDLPSLEFLDLSRNGLSFKGCCSQSDFGTTSLKYLDLSFNGVIT---   141
TLR2     FQVMKLLNQISGLLELEFDDCTLNGVGNFRASDNDRVIDPGKVETLT-IRRLHIPRFYLFYDLSTLYSLTERVKRITVE----NSKVFLVPCLLSQHLKSLEYLDLSENLMVEEYLKNSACEDAWPS--LQTLILRQNHLASLEKTG---   140
TLR5     VRHLDLSHGFVFSLNSRVFETLKDLKVLNLAYNKINKIADEAFYGLDNLQVLNLSYNLLGELYSSNFYGLPKVAYIDLQ----KNHIAIIQDQTFKFLEKLQTLDLRDNALTTIHFIPSIPDIFLSGNKLVTLPKINLTANLIHLSENRL   146
IPAF     GGLTDSLGNLKNLTKLIMDNIKMNEEDAIKLAEGLKNLKKMCLFHLTHLSDIGEGMDYIVKSLSSEPCDLEEIQLVSCC---LSANAVKILAQNLHNLVKLSILDLSENYLEKDGNEALHELIDRMNVLEQLTALMLPWGCDVQGSLS--   145
NAIP     SVDLEGNINVFSVIPEEFPNFHHMEKLLIQISAEYDPSKLVKLIQNSPNLHVFHLKCNFFSDFGSLMTMLVSCKKLTEIKFSDSFFQAVPFVASLPNFISLKILNLEGQQFPDEETSEKFAYILGSLSNLEELILPTGDGIYRVAKL---   147
```

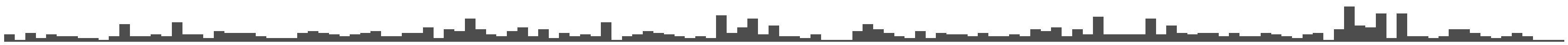

```
NOD1     ----LARALQQNTSLEILWLTQNELNDEVAESLAEMLKVNQTLKH-LWLIQNQITAK   190
NOD2     ----LALMLAKNVMLEELCLEENHLQDEGVCSLAEGLKKNSSLKI-LKLSNNCITYL   190
NOD3     ----LGQALQLNRSLTSLDLQENAIGDDGACAVARALKVNTALTA-LYLQVASIGAS   192
NOD4     ----LAESLVLCRRLEELMLGCNALGDPTALGLAQELP--QHLRV-LHLPFSHLGPG   191
NLRX1    ----LARAAREHPSLELLHLYFNELSSEGRQVLRD----------------------   159
NALP3    ----LSSVLSTNQNLTHLYLRGNTLGDKGIKLLCEGLLHPDCKLQVLELDNCNLTSH   197
NALP12   ----LSVVLQANHNLRELDLSFNDLGDWGLWLLAEGLQHPACRLQKLWLDSCGLTAK   197
RI       ----LCRVLRAKESLKELSLAGNELGDEGARLLCETLLEPGCQLESLWVKSCSFTAA   197
NALP9    ----IAAVLICNGKLKTLKLGHNEIGDTGVRQLCAALQHPHCKLECLGLQTCPITRA   197
NALP11   ----IAIVIATNEKLRSLEIGSNKIEDAGMQLLCGGLRHPNCMLVNIGLEECMLTSA   197
NALP4    ----LASALISNQNLKILQIGCNEIGDVGVQLLCRALTHTDCRLEILGLEECGLTST   196
NALP13   ----LANALSHNHNVKILDLGENDLQDDGVKLLCEALK-PHRALHTLGLAKCNLTTA   196
NALP5    ----LALALMGNSWLTHLSLSMNPVEDNGVKLLCEVMREPSCHLQDLELVKCHLTAA   197
NALP14   ----LSTSLLHNKSLTHLDLGSNWLQDNGVKLLCDVFRHPSCNLQDLELMGCVLTNA   197
NALP2    ----LTKLLQEKSSLLCLDLGLNHIGVKGMKFLCEALRKPLCNLRCLWLWGCSIPPF   197
NALP7    ----LSEALQEACSLTNLDLSINQI-ARGLWILCQALENPNCN--------------   182
NALP8    ----MISALCKNKTLKSLDLSFNSLKDDGVILLCEALKNPDCTLQILELENCLFTSI   191
TLR1     ------EMTTQMKSLQQLDISQNSVSYDEKKGDCSWTKSLLSLNMSSNILTDTIFRC   187
TLR6     ------LMTKDMPSLEILDVSWNSLESGRHKENCTWVESIVVLNLSSNMLTDSVFRC   187
TLR10    ------CFANNTP-LEHLDLSQNLLQH-KNDENCSWPETVVNMNLSYNKLSDSVFRC   187
TLR7     --NRYLQLFKNLLKLEELDISKNSLSFLPSGVFDGMPPNLKNLSLAKNGLKSFSWKK   199
TLR8     --NRYISIFKGLKNLTRLDLSLNRLKHIPNEAFLNLPASLTELHINDNMLKFFNWTL   200
TLR9     --DLYLHFFQGLSGLIWLDLSQNRLHTLLPQTLRNLPKSLQVLRLRDNYLAFFKWWS   198
NALP1    --LKLLDVSKIFPIAEIAEESSPEVVPVELLCVPSPASQGDLHTKPLGTDDDFWGPT   200
CIITA    ----LARVLPDMVSLRVMDVQYNKFTAAGAQQLAASLRRCPHVET-----LAMWTPT   177
TLR3     ------SPFQPLRNLTILDLSNNNIANINDDMLEGLEKLEILDLQHNNLARLWKHAN   198
NALP6    ----LQAVKRAKPDLVITHPALDGHPQPPKELISTF---------------------   175
TLR4     ----MSSNFLGLEQLEHLDFQHSNLKQMSEFSVFLSLRNLIYLDISHTHTRVAFNGI   194
TLR2     ------ETLLTLKNLTNIDISKNSFHSMPETCQWPEKMKYLNLSSTRIHSVTGCIPK   191
TLR5     ENLDILYFLLRVPHLQILILNQNRFSSCSGDQTPSENPSLEQLFLGENMLQLAWETE   203
IPAF     ---SLLKHLEEVPQLVKLGLKNWRLTDTEIRILGAFFGKNPLKNFQQLNLAGNRVSS   199
NAIP     ----IIQQCQQLHCLRVLSFFKTLNDDSVVEIAKVAISGGFQKLENLKLSINHKITE   200
```

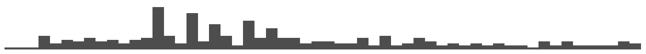

Supplement: Additional file 1 — Supplementary materials. A multiple sequence alignment used for phylogenetic analysis in Figure 1. [file 1471-2172-10-48-S1.pdf]
